# 

**Published:** 1915-10

**Authors:** 


### ANNOUNCEMENTS

CALIFORNIA REGISTRATION EXAMINATIONS. —The California State Board of Examination will be held in San Francisco, in December, 1915, and to any dentist who contemplates taking the examination and feels the need of a preparatory course. Such a course will be conducted in Los Angeles by Dr. Endelman commencing November first. Further information may be obtained by addressing Dr. J. Endelman, care of The Jas. W. Edwards Co., San Francisco, Cal.

### Circular for the Information of Persons Desiring to Enter the Dental Corps of the United States Navy.

A candidate for appointment to the Dental Corps of the Navy as acting assistant dental surgeon must be a citizen of the United States, between 24 and 32 years of age, a graduate of a standard medical or dental college trained in the several branches of dentistry, of good moral character, and of unquestionable professional repute.

Should an assistant dental surgeon, Dental Reserve Corps, desire to enter the Dental Corps, he must be between 22 and 30 years of age, a graduate of a reputable school of medicine or dentistry, of good moral character, and of unquestionable professional repute. In accordance with law, however, prior to being commissioned an assistant dental surgeon in the Dental Corps he must serve three years as an acting assistant dental surgeon, as explained below.

Successful candidates are first appointed acting assistant dental surgeons, and after serving a probationary period of three years are ordered before an examining board to determine their fitness for commission as assistant dental surgeons, United States Navy.

Application should be made to the Chief of the Bureau of Navigation, Washington, D. C., via. the Surgeon General, United States Navy, and according to the form prescribed. This application *must be in the handwriting of the candidate* and must be accompanied by the following certificates:

(*a*) Letters or certificates from two or more persons of good repute, testifying from personal knowledge to good habits and moral character.

(*b*) A certificate to the effect that the applicant is a citizen of the United States.

(*c*) Certificate of preliminary education. The candidate must submit a certificate of graduation from an accepted high school or an acceptable equivalent.

(*d*) Certificate of dental education. This certificate should give the name of the school and the date of graduation.

(*e*) If the candidate has had special educational or professional advantages, certificates to this effect, signed by the proper authorities, should also be forwarded.

The applicant will save unnecessary correspondence if he will make sure when submitting his application that the qualifications enumerated above are clearly and plainly described in his letters or certificates.

### Form of Application.

(This form is not to be filled in here, but copied on a separate sheet in the handwriting of the applicant.)

..............................

.............................., 191

SIR: I request permission to be examined for an appointment as acting assistant dental surgeon in the United States Navy. I was born at ........., and was ....years of age on the ...... day of ........., 191..; am a citizen of the United States, residing in .........., county of .........., in the state of ......... I am a graduate of ......... dental (medical) school, in the State of ........., and was licensed to practice dentistry in the State of ......... in .........

I forward herewith letters testifying to my moral character, habits, citizenship, preliminary education, professional education, and qualifications.

Very respectfully,

..............................

The CHIEF OF THE BUREAU OF NAVIGATION,
*Navy Department, Washington, D. C.* (via. The Surgeon General, United States Navy).

A candidate whose qualifications are satisfactory will receive a formal permit to present himself for examination.

THE EXAMINATION.

When a candidate presents himself for examination he must bring with him the testimonials as to character and professional fitness, diploma of preliminary and professional education, and a certificate that he is a citizen of the United States; those forwarded with his application being returned to him for this purpose.

The examination is conducted in the following order: I. Physical. II. Professional. III. Collateral.

*I. Physical Examination.*

The physical examination is thorough, and the candidate is required to certify on oath that he is free from all mental, physical, and constitutional defects. (Applicants are advised to carefully read the special circular regarding physical qualifications inclosed.)

Acuteness of vision, 12-20 for each eye, unaided by glasses, but capable of correction, by aid of lenses, to 20-20 is obligatory. Color perception must be normal and the teeth good.

If the candidate is found to be physically disqualified, his examination is concluded; if found to be physically qualified, his examination is continued as follows:

### *II. Professional Examination.*

1. Letter to the board describing in detail his general and professional education.
2. Tests of skill in practical dentistry.
3. Proficiency in the several usual subjects in a standard dental college course.

Theoretical (Written and Oral).

Anatomy, physiology, physics, chemistry, metallurgy, dental materia medica and therapeutics, dental pathology and bacteriology, orthodontia, oral surgery, operative dentistry (theory), and prosthetic dentistry (theory).

Practical (Clinical).

Operative dentistry and prosthetic dentistry.

### *III. Collateral Examination.*

This examination is oral, and is conducted in the following subjects: Arithmetic, grammar, general history, physics, general literature, and Latin.

Applicants holding diplomas or certificates from reputable literary or scientific colleges, normal schools, or high schools may submit such diplomas or certificates for the consideration of the board in this connection.

A successful candidate, upon completion of his examination, will be notified by the president of the board that he has been found qualified.

With the consent of the board, a candidate may withdraw at any period from further examination and may at a future time present himself for re-examination. The board may conclude the examination (written, oral, and practical) at any time, and may deviate from this general plan as it may deem best for the interests of the naval service.

*No allowance will be made for the expense of persons appearing for examination.*

The tenure of office in the Dental Corps of the Navy except in the case of acting assistant dental surgeons appointed for temporary service only, is for life, unless sooner terminated by removal, resignation, disability, or other casualty.

All officers of the Dental Corps are retired from active service at the age of 64 years, and when so retired (or when retired from active service for disability or other casualty contracted in the line of duty before that age) receive an annual pay for life amounting to three-fourths of the pay of the grade or rank held by them in the time of retirement, including the increased pay allowed for length of service as explained below.

When an officer of the Navy, including dental officers, has been 30 years in the service, he may, upon his own application, in the discretion of the President, be retired from active service and placed upon the retired list at an annual pay for life amounting to three-fourths of the pay of the grade or rank held by him at the time of retirement, including the increased pay allowed for length of service.

Immediately upon official notification of the death from wounds or disease not the result of his own misconduct of any officer of the Navy, the Paymaster General of the Navy shall cause to be paid to the widow, and if no widow, to the children, and if there be no children, to any other dependent relative of such officer previously designated by him, an amount equal to six month's pay at the rate of pay received by such officer at the date of his death, less $75 to defray expenses of interment; and the residue, if any, of the amount reserved shall be paid subsequently to the designated person. No deduction shall be made on account of expenses of preparation or transportation of the remains.

When traveling under orders by other than public conveyance, officers of the Navy, including dental officers, receive 8 cents a mile to defray the expenses of such travel performed from point to point within the United States, and when so traveling abroad are allowed actual personal expenses estimated on a liberal basis in accord with the position of the officer, both as regards admissible items of expense and the cost of such items.

For every five years' service the pay of officers is increased 10 per cent. (though not to exceed 40 per cent), calculated on the annual base pay of their grade, as shown in appended table.

When an officer goes to sea or leaves the continental limits of the United States under assignment to stations or for the performance of other duties beyond the seas, his pay is increased 10 per cent.

When two or more candidates are examined at the same time, their appointments will be in order of merit reported by the board.

Officers of the Dental Corps have the rank of lieutenant (junior grade), and are entitled to all the military courtesies and consideration that go with that rank and are accorded to officers of other branches of the service in a similar grade. They wear the same uniform as other officers of the Navy, with a designating device distinctive of their corps.

*Pay and Allowance Table.*

| Length of service. | Pay per annum on shore. | Allowances per annum for quarters. | Total pay and allowances per annum on shore. | Pay per annum at sea. |
|---|---|---|---|---|
| First five years' service...... | $2,000 | $432 | $2,432 | $2,200 |
| After five years' service..... | 2,200 | 432 | 2,632 | 2,420 |
| Aften ten years' service..... | 2,400 | 432 | 2,832 | 2,640 |
| After fifteen years' service.. | 2,600 | 432 | 3,032 | 2,860 |
| After twenty years' service.. | 2,800 | 432 | 3,232 | 3,080 |

A limited number of acting assistant dental surgeons are authorized by law for temporary appointment when their services are required. Their pay and allowance are the same as those of acting assistant dental surgeons in the regular service. The law provides that these temporary appointments may be revoked at any time, shall have no legal force or effect .except for the time the temporary appointee is in active service, and shall include no right to retirement. The requirements for appointment are similar in general to those outlined above. Application for permission to take the examination should be addressed to the Chief of the Bureau of Navigation, Navy Department, Washington, D. C., via. the Surgeon General, following the form given above.

For further information address the Surgeon General, United States Navy, Navy Department, Washington, D. C.

